# Cardiac cephalalgia-headache as an atypical presentation of ST-segment elevation myocardial infarction: a case report

**DOI:** 10.1186/s12872-025-04898-z

**Published:** 2025-06-07

**Authors:** Udayanga Andadola, Subhani Poornima, Gamini Galappatthy

**Affiliations:** 1 Department of Primary Care and Family Medicine, Faculty of Medicine, Sabaragamuwa University of Sri Lanka, Ratnapura, Sri Lanka; 2 Cardiology Unit, The National Hospital of Sri Lanka, Colombo, Sri Lanka

**Keywords:** Case report, Cardiac cephalalgia, Acute coronary syndrome, Coronary angiography, Diabetes mellitus

## Abstract

**Background:**

Ischaemic heart disease commonly presents with chest pain and autonomic symptoms; however, atypical manifestations can occur. Cardiac cephalalgia is a rare presentation of acute coronary syndrome, characterised by a migraine-like headache triggered by myocardial ischaemia. Diagnosis requires a high index of suspicion.

**Case presentation:**

We describe a 47-year-old man with diabetes and a history of smoking who presented with an acute, severe frontotemporal headache accompanied by nausea and vomiting. Electrocardiography revealed ST-segment elevation in the inferior leads. Coronary angiography demonstrated multivessel coronary artery disease involving the right coronary artery and the left anterior descending artery. A subsequent measurement of serum troponin I confirmed myocardial injury. Both arteries were successfully stented, leading to clinical improvement and resolution of the headache.

**Conclusions:**

This case highlights the importance of considering cardiac causes in patients presenting with severe headaches particularly in those with cardiovascular risk factors.

**Supplementary Information:**

The online version contains supplementary material available at 10.1186/s12872-025-04898-z.

## Introduction

Coronary heart disease remains a leading cause of global morbidity and mortality. In 2020, an estimated 244.1 million individuals were living with ischaemic heart disease, with a higher prevalence among males than females [1]. Typical symptoms of myocardial ischaemia include chest discomfort, often radiating to the upper extremities, mandible, or epigastrium, and may occur at rest or during exertion. However, atypical presentations such as palpitations or even silent ischaemia are also recognised [2]. Cardiac cephalalgia is a rare but important atypical manifestation of myocardial ischaemia, characterised by headache resembling a migraine, which occurs during episodes of myocardial ischaemia [3–11]. Due to its rarity and non-specific presentation, the diagnosis may be delayed or overlooked, particularly in the absence of chest pain.

In this report, we present a case of inferior ST-segment elevation myocardial infarction (STEMI) in a middle-aged man who presented solely with headache, emphasising the importance of recognising cardiac cephalalgia as a potential manifestation of acute coronary syndrome (ACS).

## Case presentation

A 47-year-old male driver with a 15-year history of diabetes mellitus and a 20-year history of smoking approximately five pack-years, presented to the emergency treatment unit of a tertiary care hospital in Colombo, Sri Lanka, with a severe, generalised headache lasting four hours. The headache had awakened him from sleep and was most intense in the frontotemporal region. It was described as continuous, tightening in nature, and associated with nausea, two episodes of vomiting, and feeling of faint. He denied photophobia, phonophobia, chest pain, arm or neck pain, dyspnoea, or diaphoresis.

At home, he self-administered 1 g of paracetamol and applied an Ayurvedic balm to the forehead without symptom relief. Due to the persistence of symptoms, he sought medical care.

Two days prior, he had experienced mild fever and throat irritation, without headache, and was prescribed paracetamol (1 g) as needed, diclofenac sodium (50 mg) twice daily, amoxicillin (500 mg) three times a day, and omeprazole (20 mg) twice daily, by a private clinic. His fever and throat symptoms had resolved by the day before presentation, which he spent resting.

His diabetes was initially managed with metformin, later transitioned to Mixtard 30 insulin over the preceding four years. He was poorly adherent to therapy and had irregular clinic follow-up, often procuring medications over the counter. Screening for diabetic complications had not been performed. His past surgical history included incision and drainage of abscesses on the left leg and right groin. Both parents had diabetes, and his father had died from complications of alcoholic cirrhosis.

**Fig. 1:**
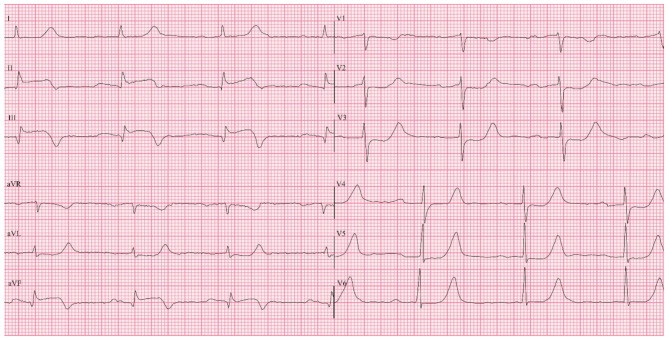
Initial ECG upon admission: note the ST segment elevations in leads II, III, aVF, and mild degree ST segment depressions in leads V2 - V4

On admission, he was afebrile and hemodynamically unstable, with a pulse rate of 50 bpm and blood pressure of 88/50 mmHg. Oxygen saturation on room air was 100%. Cardiovascular and respiratory examinations were unremarkable. Neurological examination was normal. Electrocardiography (ECG) revealed ST-segment elevation in leads II, III, and aVF, with reciprocal ST depression in leads V2–V4 (Fig. 1). A diagnosis of inferior STEMI with first-degree atrioventricular block was made.

He received loading doses of aspirin (300 mg), clopidogrel (300 mg), and atorvastatin (80 mg), along with atropine (1.2 mg in divided doses) and an isoprenaline infusion (5 µg/min). Approximately 30 min after initial treatment, the headache began to improve.

Upon transfer to the cardiology unit of the National Hospital of Sri Lanka, he was alert and oriented. His pulse was 84 bpm, respiratory rate 20 bpm, blood pressure 100/60 mmHg, and oxygen saturation 98% on room air. The isoprenaline infusion was continued, and insulin therapy was optimised.

**Fig. 2:**
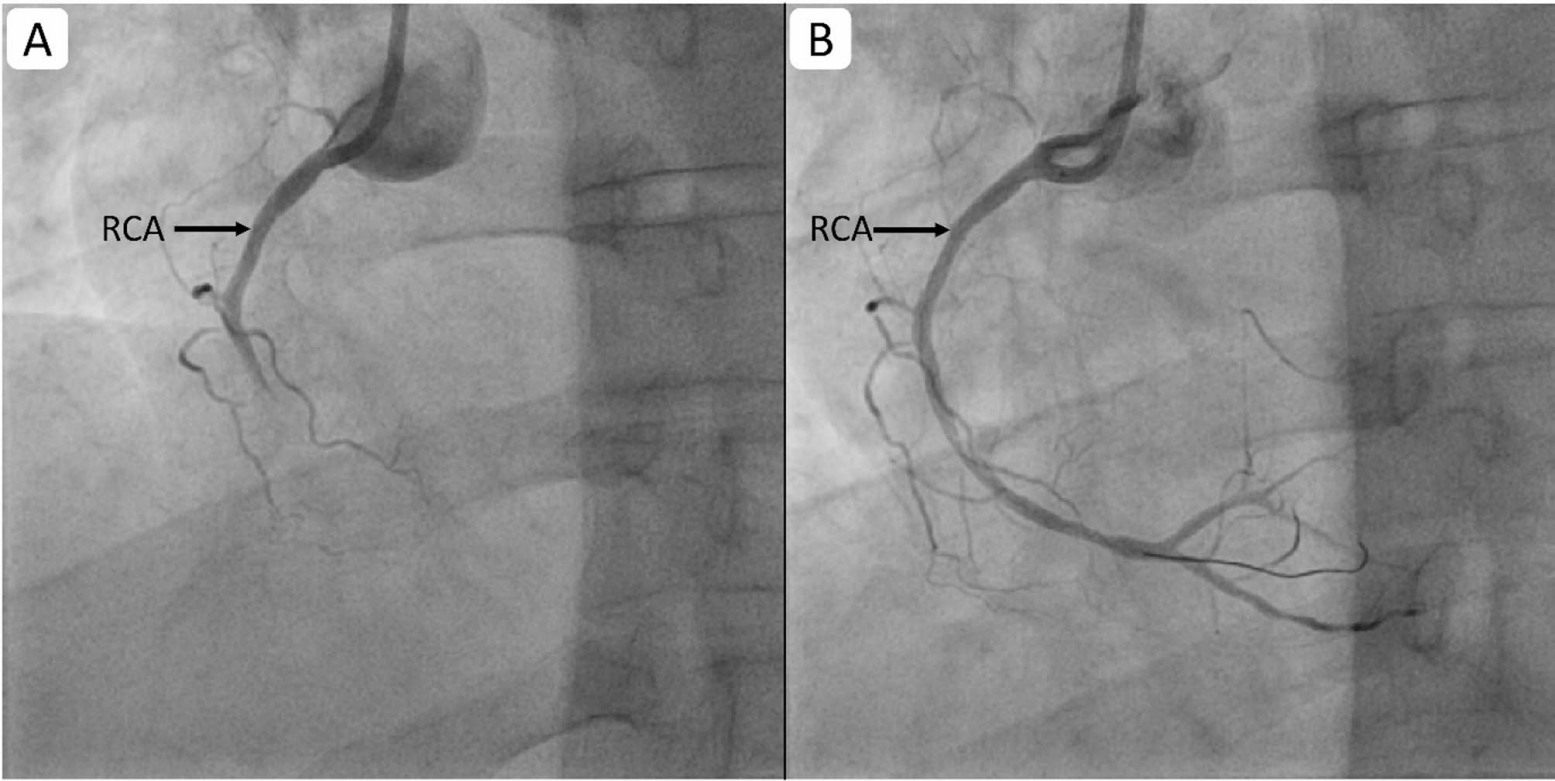
Coronary angiogram showing RCA before (**A**) and after (**B**) stenting: Note the recanalized RCA after stenting (RCA: Right coronary artery)

**Fig. 3:**
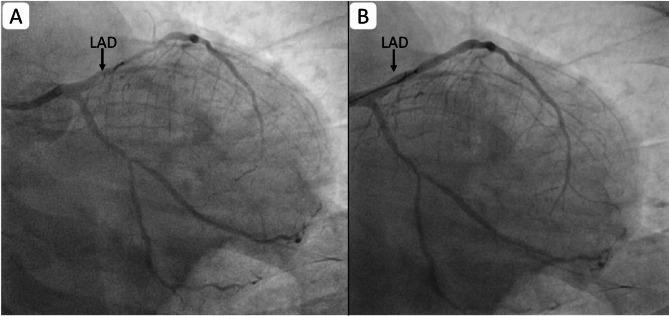
Coronary Angiogram Showing LAD before (**A**) and after (**B**) stenting: Note the recanalized dialated LAD after stenting (LAD: Left anterior descending artery)

**Fig. 4:**
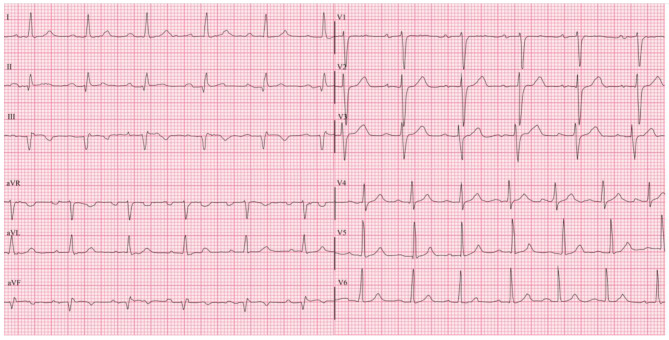
Post percutaneous coronary intervention ECG: note the post-stenting resolution of ST elevations and depressions

Coronary angiography revealed total thrombotic occlusion of the mid-right coronary artery (RCA) [Figure 2(A)] and significant long-segment stenosis, up to 80% of the proximal to mid-left anterior descending artery (LAD) [Figure 3(A)], confirming multivessel coronary artery disease. Both vessels were successfully stented [Figures 2(B) and 3(B)]. Following revascularisation, the patient’s headache resolved completely, and the ECG normalised [Figure 4]. Isoprenaline was gradually withdrawn.

Transthoracic echocardiography demonstrated an ejection fraction of 55%, with regional wall motion abnormality in the inferior wall, and no pericardial effusion.

Laboratory investigations revealed a markedly elevated serum troponin I level of 6.14 ng/mL (reference: 0.00–0.04 ng/mL). His serum creatinine on admission was elevated at 1.85 mg/dL (reference: 0.72–1.25 mg/dL), which improved to 1.1 mg/dL on day two, suggesting acute kidney injury likely due to dehydration and renal hypoperfusion. Aspartate aminotransferase and alanine aminotransferase levels were also elevated (128 U/L and 123 U/L, respectively) but decreased to 38 U/L and 65 U/L on day two. Abdominal ultrasound was unremarkable.

The patient was discharged three days later on dual antiplatelet therapy (aspirin and clopidogrel), atorvastatin, bisoprolol, enalapril, and Mixtard 30 insulin, with outpatient follow-up arranged. At the two-week review and subsequent monthly follow-ups, he remained asymptomatic and had resumed his routine occupational activities.

## Discussion

ACS classically present as a tightening-type chest pain on the left side, which may radiate to the neck, jaw, or upper limbs. However, atypical presentations are not uncommon, and diagnosing myocardial ischaemia in such scenarios requires a high degree of clinical suspicion. A thorough history and physical examination remain vital in identifying ACS, especially in patients with risk factors such as diabetes mellitus [12].

This case aligns with previously reported instances of cardiac cephalalgia, where headache was the primary symptom of acute coronary events. In a case series by Kobata et al., four patients exhibited similar presentations, with headaches preceding or coinciding with myocardial ischaemia. Notably, common cardiovascular risk factors such as hypertension, diabetes, hyperlipidaemia, and smoking were prevalent among these patients.

In a review of 30 cases, Wei and Wang found that patients commonly presented with migraine-like headaches, often located in the frontotemporal or occipital regions. In some cases, headache was the sole manifestation of ischaemia. The majority of patients were males over 50 years of age with typical cardiovascular risk factors. Headaches were frequently associated with autonomic symptoms, and electrocardiographic changes along with elevated cardiac enzymes supported the diagnosis. Importantly, symptom resolution followed appropriate coronary intervention in most cases.

Our patient presented with a sudden-onset severe headache that awakened him from sleep, an unusual manifestation of ACS [13]. Although subarachnoid haemorrhage (SAH) is the most common life-threatening cause of sudden-onset severe headache, myocardial ischaemia should also be considered, particularly when the risk factors for ischaemic heart disease are present and typical intracranial causes are excluded. Ideally, a non-contrast CT brain should be the first step in excluding SAH [14]. However, in this patient, the presence of ECG changes and haemodynamic instability pointed to a diagnosis of STEMI, indicating that an urgent coronary intervention was necessary. The severity and onset of the headache ruled out a post-febrile headache, while the absence of focal neurological deficits, in combination with the ECG findings, supported the diagnosis of cardiac cephalalgia [15].

Cardiac cephalalgia is a rare presentation of myocardial ischaemia [3–11, 16]. According to the International Classification of Headache Disorders (ICHD-3), cardiac cephalalgia is defined as a migraine-like headache that is usually, but not always, triggered by exertion, occurs in the context of myocardial ischaemia, and is relieved by nitrates [15]. The diagnostic criteria for cardiac cephalalgia are summarised in [Table 1].

**Table 1:** Diagnostic criteria for cardiac cephalalgia

Diagnostic criteria for cardiac cephalalgia according to International Classification of Headache Disorders-3 (ICDH-3)
A. Any headache fulfilling criterion C
B. Acute myocardial ischaemia has been demonstrated
C. Evidence of causation demonstrated by at least two of the following:
1. Headache has developed in temporal relation to the onset of acute myocardial ischaemia
2. Either or both of the following:
a) Headache has significantly worsened in parallel with worsening of the myocardial ischaemia
b) Headache has significantly improved or resolved in parallel with improvement in or resolution of the myocardial ischaemia
3. Headache has at least two of the following four characteristics:
a) Moderate to severe intensity
b) Accompanied by nausea
c) Not accompanied by photophobia or phonophobia
d) Aggravated by exertion
4. Headache is relieved by nitroglycerine or derivatives of it
D. Not better accounted for by another ICHD-3 diagnosis.

*Note: Table reproduced under the copyright permission of the Headache Classification Committee of the International Headache Society

This patient had several risk factors for coronary artery disease, including long-standing poorly controlled diabetes mellitus and a significant smoking history. Diabetes is associated with accelerated atherosclerosis and autonomic neuropathy, which may blunt the typical anginal symptoms and result in silent or atypical presentations such as fatigue, dyspnoea, nausea, abdominal pain, or rarely, headache [17].

Autonomic neuropathy leads to sensory denervation of the cardiac afferents, which may explain the absence of chest pain in diabetic patients with myocardial ischaemia. Histological studies have shown abnormalities in autonomic nerve fibres in such patients [18]. Imaging studies like m-Iodobenzylguanidine scintigraphy also support the theory of sympathetic denervation contributing to altered pain perception in diabetic patients [19]. Furthermore, the lack of a circadian pattern in myocardial events in diabetics suggests significant autonomic dysregulation [20, 21].

Distinguishing cardiac cephalalgia from migraine is particularly crucial. Triptans and ergot derivatives, often used to treat migraine, are vasoconstrictors and contraindicated in patients with myocardial ischaemia [15]. Hence, it is prudent to include ECG in the initial evaluation of patients presenting with acute headache, particularly in those with cardiovascular risk factors. Serial ECGs and cardiac biomarkers may further aid diagnosis [22].

The underlying pathophysiology of cardiac cephalalgia is not fully understood [7, 10, 11, 23]. However, several hypotheses have been proposed to elucidate its mechanism. One hypothesis involves the referred pain from convergence of afferent cardiac vagal fibres and afferent pathways of cranial pain fibres [7, 11, 24]. Another proposed mechanism implicates the release of proinflammatory neurotransmitters such as bradykinin, histamine, adenosine, and serotonin, during myocardial ischaemia, which may lead to cerebral vasodilation and subsequently provoke headache [24, 25]. A further theory suggests that headache may result from increased venous congestion as a consequence of acute heart failure following myocardial ischaemia [26].

In this patient, the referred pain mechanism seems plausible. His longstanding poorly controlled diabetes likely resulted in cardiac autonomic neuropathy, masking typical ischaemic pain while allowing alternative afferent pathways to mediate headache [27].

Interestingly, he had a recent history of upper respiratory tract infection (URTI) with throat irritation. URTIs are recognised triggers for ACS, particularly in the first two weeks following infection [28, 29]. The proposed mechanism involves inflammatory and pro-thrombotic processes that destabilise atherosclerotic plaques, increasing the risk of acute coronary events [28]. The patient had also received diclofenac sodium (50 mg twice daily), a non-steroidal anti-inflammatory drug (NSAID), for two days prior to admission. NSAIDs, especially diclofenac, are associated with increased cardiovascular risk and may precipitate ACS, particularly in patients with pre-existing cardiovascular risk factors [30–32].

## Conclusion

Although headache is a rare symptom of acute myocardial ischaemia, cardiac cephalalgia is a recognised but underdiagnosed entity. Clinicians should consider ACS in the differential diagnosis when a patient, especially one with risk factors such as diabetes or smoking, presents with a new-onset severe headache without neurological deficits or prior history of migraine. A simple ECG may be lifesaving in such cases. In high-risk patients, serial ECGs and cardiac biomarker testing should be considered. Increased awareness and early recognition of cardiac cephalalgia can prevent misdiagnosis and enable timely management of potentially fatal cardiac events.

## Electronic supplementary material

Below is the link to the electronic supplementary material.

Supplementary Material 1

## Data Availability

No datasets were generated or analysed during the current study.

## Abbreviations

ACS: Acute coronary syndrome
ECG: Electrocardiogram
ICDH: International Classification of Headache Disorders
IHS: International Headache Society
LAD: Left anterior descending artery
NSAID: Non-steroidal anti-inflammatory drugs
RCA: Right coronary artery
SAH: Subarachnoid haemorrhage
STEMI: ST segment elevation myocardial infarction
URTI: Upper respiratory tract infection
