# Supplementary material for: Cardiac cephalalgia-headache as an atypical presentation of ST-segment elevation myocardial infarction: a case report
Source: BMC Cardiovasc Disord. 2025 Jun 7;25:435. doi: 10.1186/s12872-025-04898-z (PMC12145586; doi:10.1186/s12872-025-04898-z)
Supplement: Supplementary file 1 — Supplementary Material 1 [file 12872_2025_4898_MOESM1_ESM.pdf]

CARE case report guidelines

CARE Checklist of information to include when writing a case report

(cc) BY-NC-ND

Topic	Item	Checklist item description	Reported on Line
Title	1	The diagnosis or intervention of primary focus followed by the words "case report"	16, 17
Key Words	2	2 to 5 key words that identify diagnoses or interventions in this case report, including "case report"	35
Abstract (no references)	3a	Introduction: What is unique about this case and what does it add to the scientific literature?	19
	3b	Main symptoms and/or important clinical findings	25, 26
	3c	The main diagnoses, therapeutic interventions, and outcomes	27, 28, 29, 30
	3d	Conclusion—What is the main "take-away" lesson(s) from this case?	31
Introduction	4	One or two paragraphs summarizing why this case is unique (**may include references**)	36
Patient Information	5a	De-identified patient specific information.	50
	5b	Primary concerns and symptoms of the patient.	52
	5c	Medical, family, and psycho-social history including relevant genetic information	62- 66
	5d	Relevant past interventions with outcomes	-
Clinical Findings	6	Describe significant physical examination (PE) and important clinical findings.	67- 69
Timeline	7	Historical and current information from this episode of care organized as a timeline	-
Diagnostic Assessment	8a	Diagnostic testing (such as PE, laboratory testing, imaging, surveys).	69- 71, 80- 82, 96- 101
	8b	Diagnostic challenges (such as access to testing, financial, or cultural)	-
	8c	Diagnosis (including other diagnoses considered)	71, 82
	8d	Prognosis (such as staging in oncology) where applicable	84
Therapeutic Intervention	9a	Types of therapeutic intervention (such as pharmacologic, surgical, preventive, self-care)	72- 73, 80- 83
	9b	Administration of therapeutic intervention (such as dosage, strength, duration)	72- 73, 102- 103
	9c	Changes in therapeutic intervention (with rationale)	-
Follow-up and Outcomes	10a	Clinician and patient-assessed outcomes (if available)	94- 95
	10b	Important follow-up diagnostic and other test results	-
	10c	Intervention adherence and tolerability (How was this assessed?)	80- 84
	10d	Adverse and unanticipated events	-
Discussion	11a	A scientific discussion of the strengths AND limitations associated with this case report	123- 131, 164- 166
	11b	Discussion of the relevant medical literature **with references**.	213
	11c	The scientific rationale for any conclusions (including assessment of possible causes)	156- 173
	11d	The primary "take-away" lessons of this case report (without references) in a one paragraph conclusion	175- 181
Patient Perspective	12	The patient should share their perspective in one to two paragraphs on the treatment(s) they received	-
Informed Consent	13	Did the patient give informed consent? Please provide if requested	Yes ☑ No ☐
